# How do young children respond to the distress of others? Applying infrared thermography and behavioural analyses to examine the development of emotion contagion and empathy

**DOI:** 10.1371/journal.pone.0335537

**Published:** 2026-03-04

**Authors:** Diane A. Austry, Elizabeth Renner, Zanna Clay

**Affiliations:** 1 Department of Psychology, Durham University, Durham, United Kingdom; 2 School of Psychology, Northumbria University, Newcastle-upon-Tyne, United Kingdom; Autonomous University of Queretaro: Universidad Autonoma de Queretaro, MEXICO

## Abstract

Empathy is a core feature of the human social experience, underpinned by the sharing of and understanding of others’ states. However, we know relatively little about its early development. Here, we used the emerging technique of infrared thermography, combined with behavioural observations, to investigate how young children (1–3 years old) respond to others’ distress. We measured the emotional reactions – including changes in facial skin temperature and behavioural responses – of N = 30 typically developing children from British nurseries while they watched short video-vignettes of familiar and unfamiliar caregivers displaying emotional distress or neutral expressions. We hypothesised that children’s facial temperature change and behavioural responses (including facial expressions and self-directed behaviours) would be stronger to distress than to neutral stimuli. Based on the social bias of empathy, we hypothesised that responses would be stronger towards familiar individuals as compared to unfamiliar. Results confirmed that children’s thermal response at the nose bridge was stronger when observing someone in distress compared to the neutral control. Although model familiarity had only a weak effect, there was an effect of age, with younger infants showing stronger thermal responses to distress stimuli compared to older children. We found no effect on behavioural responses. We discuss implications of these results, including whether thermal-imagery may be a more sensitive measure of affective responding than behavioural measures, and the importance of using multiple methods. By combining physiological and behavioural measures, this study contributes to theoretical and methodological advances into understanding how internal affective processes map onto external measures in early childhood, shedding light on the development of empathy.

## Introduction

Empathy is a core feature of human social functioning, consisting of *affective* (sharing others’ emotional states) and *cognitive* components (understanding those states) [1,2]. Recent research has suggested that, even in the first year of life, infants are capable of empathically responding to others’ distress, with both affective and cognitive components of empathy in place to some degree [3,4,5]. Davidov and colleagues [6] found that infants as young as 3 months show concerned facial affect and exploration of the other’s distress, seen as evidence for empathic responding. Furthermore, early expressions of empathy in this sample predicted prosocial comforting behaviours at 18 months. Overall, the emerging data suggest that the capacity for concern and the motivation to understand others’ emotions are present very early in human ontogeny.

To respond empathically, one needs to be sensitive to others’ states and to perceive their emotional expressions. In the first year of life, hearing the crying of another infant is known to initiate ‘contagious crying’ [7,8,9,10]. This form of emotion contagion has been interpreted as *personal distress,* suggesting that infants experience another’s distress as their own and are unable to separate another’s state from their own [11,12]. However, one study found that, upon hearing a crying peer, most 6-month-old infants showed other-oriented responses as compared to self-focused reactions, which included looking toward the infant in distress; some also gestured towards them or touched them, to communicate [13]. Similar findings have also been demonstrated more recently in 10-month-old infants, where personal distress to hearing peer distress was rare [14]. Beyond the first year of life, studies have focused on infant and toddler responses to the distress of others [e.g., 15,16,5]; the affective component is thought to be reflected by facial expressions, vocalizations, and gestures of concern, while the cognitive component is thought to refer to the child’s inquiry, or hypothesis testing behaviour.

Infra-Red Thermography (IRT) represents a novel technology that has recently emerged in the field of emotion research. IRT records the thermal infrared signals emitted by the body and allows the measurement of the changes in skin temperature which result from changes in blood pressure activated by sympathetic nerves [17]. In this sense, IRT provides an indicator of autonomic nervous system activity which is implicated in emotion contagion [18]. Empirical research in humans has demonstrated that emotion-based states induce distinctive changes in facial skin temperature [19], and such changes have been recently reported in great apes [20,21,22]. Although it has received minimal application as yet, IRT can be a valuable tool to use in research with young children as it is non-invasive and contact-free [14]. This contrasts to measures such as heart rate, respiratory sinus arrhythmia, and skin conductance [23] which have also been suggested as relevant markers of empathy [24,25,26,27] but are either restricted to artificial settings or require some degree of contact [23].

The overall aim of the current study was to investigate the development of empathy using the emerging technique of IRT combined with behavioural measures. Simultaneously examining both inner arousal and external behavioural components allows for a more systematic investigation of the coordinated changes taking place during an emotional response. As behaviour and physiology may give differing insights, it is imperative to examine emotionality via a range of measures to understand the scope of its complexity. We presented young children with a series of empathy-eliciting video vignettes that showed familiar or unfamiliar women either in distress or in a neutral pose. To examine the developmental trajectory, we also looked at whether behavioural and physiological responses changed across age from 1 to 3 years.

Based on the assumption of eliciting emotion contagion, we predicted that children’s thermal and behavioural responses would be stronger when viewing another’s distress versus neutral expressions. Given that previous research has reported both increases and decreases in facial temperature in response to affective stimuli [e.g., Ioannou et al., 2013;[28] 29,30,14], our hypotheses focus on magnitude of temperature change, rather than direction. That is, we predicted that absolute facial temperature changes from baseline would be larger in the distress condition than in the neutral condition, and that children would produce more facial expressions typical of negative situations (such as concern, discomfort, or distress). According to the *empathy gradient* hypothesis, emotional responses to others’ states is strongest among kin, followed by socially close partners, and weakest among socially distant or unfamiliar individuals [31,32,33,34]. We therefore predicted that children’s thermal and behavioural responses would be stronger in response to a familiar individual showing distress as compared to an unfamiliar individual. We did not predict such differences when the stimulus was of neutral valence (familiarity * valence interaction).

Self-directed behaviours are used as a behavioural marker of stress and emotional arousal in both human and non-human primates [e.g., self-scratching, 35,36,37,38,39,40], for example when observing a conflict [41]. Therefore, we predicted that children would show enhanced arousal in response to distress over neutral expressions, corresponding to more self-directed behaviours. Avoidance behaviours can also offer insights into whether the elicited response remains as self-oriented personal distress or other-oriented empathic responding. This is because unlike the more basic mechanisms of emotion contagion, which can be self or other-oriented, empathic responding relies on orienting to the individual in distress [2]. In this regard, a higher occurrence of avoidance-based behaviours would be indicative of self-distress rather than empathy [25]. Therefore, we predicted that self-distress (as measured via self-directed behaviours) would result in greater avoidance behaviours during the distress stimuli than the neutral ones, and that younger children would react with more self-distress and fewer markers of concern than the older ones, therefore showing less avoidance overall.

Finally, we also examined potential sex differences. Neurological studies show gender effects on emotional responding in adults, with greater neural reactivity to emotional stimuli in women compared to men, particularly for negative stimuli [42,43]. Women also display a different physiological response (i.e., the pattern of skin conductance) compared to men when watching emotional video clips [44]. During childhood, boys show lower inhibitory control than girls [45], therefore we predicted that boys would react more strongly to the affective stimuli, indicated by larger temperature changes from baseline as well as more self-directed behaviours and expressions of facial concern in boys compared to girls. Alternatively, if females are more behaviourally responsive to others’ emotions than males [46,47,48,49,44,50], there may be a behaviour-thermal discontinuity, whereby girls will be more behaviourally responsive than boys to the distress condition. Furthermore, because girls score higher in seeking social support than boys, and boys score higher in avoidant coping [51], we predicted sex differences in behavioural responses to distress stimuli, with girls showing enhanced communicative responding and information-seeking behaviours.

By integrating physiological and behavioural indices, the goal of this study was to go beyond traditional observation-only approaches by detecting underlying empathic arousal regardless of outward behaviour, thereby reducing the risk of underestimating children’s affective sensitivity. Collectively, our approach aims to advance understanding of the mechanisms underpinning empathy development, via a multi-method approach, and to probe new methodological avenues for investigating how everyday exposure to others’ emotions shapes young children’s social and emotional outcomes.

## Materials and methods

### Ethics statement

This study was approved by the Psychology Sub-Ethics Committee at Durham University, which includes full compliance to the General Data Protection Regulation Act (GDPR). We collaborated with the nursery managers to contact and inform the parents. We obtained written informed consent in the form of a signed checklist from the parents to include their child in the study. Before conducting the experiment, we also obtained the child’s verbal assent and stopped a trial if the child requested (verbally or behaviourally). The caregivers who agreed to be recorded to create the emotional stimuli also provided informed consent using written documentation. The study was conducted with full compliance with the ethical standards of the British Psychological Society. Participant confidentiality was safeguarded by assigning anonymous participant codes, and all datafiles were stripped of direct identifiers before analysis. Data were stored on encrypted, password-protected servers accessible only to the authorised research team. Nurseries are referred to only by broad geographic region, ensuring that participants nor settings can be identified. The individuals/participants who feature within the Figure images and the Supporting Information S1–S3 Videos in this manuscript have provided written informed consent (as outlined in PLOS consent form) to publish these excerpt image details alongside the manuscript.

### Participants

Typically developing children were recruited from two day-care nurseries in the County of Durham, UK between 04.04.2019–17.05.2019. The nurseries were selected for inclusion based on convenience sampling, with additional practical criteria ensuring suitability for data collection. Specifically, their willingness to participate, proximity to the research institution, and the availability of quiet, enclosed indoor spaces with stable lighting conditions, essential for reliable infrared thermography and behavioural observation. Both nurseries served demographically diverse populations and followed standard early years curricula, supporting ecological validity within typical childcare environments. According to the Office for National Statistics, the demographics of the area are primarily white, Christian, and with an average annual gross earning of £22,188 in 2020 [52].

A total of 39 children participated in the study; data from N = 30 children were included in the analyses (age range = 1.1 to 3.0 years old; mean = 2.0 years old; SD = 0.5; 17 girls; see S1 Table in S1 File). Data from some children were excluded for the following reasons: data from three children were excluded because of the low quality of the data collected (e.g., child movement and lack of attention towards the stimulus); one due to the child falling asleep during the trial; two due to the children requesting to leave after showing lack of attention and boredom; and three because they were new at the nursery and thus were evaluated to not have had time to develop a strong enough social relationship with their caregivers for this study (but who took part upon their request). One child showed signs of discomfort (seemingly shy) during the acclimatisation phase, even after spending a lot of time with the experimenter, so we did not proceed to data collection in this case. Sample size was determined by balancing the need for sufficient data to detect a signal using thermographic methods and the resources available.

In both nurseries, each child had a ‘key carer’ with whom they developed a strong social relationship. This person spent more quality time with them, including one-to-one interactions (e.g., play or reading sessions). Given this social bond, we used this key person wherever possible as the familiar stimulus for our experiment (see below). All trials were conducted between 9am–12pm in April-June 2019.

### Study design

This study employed a two-factor within-subjects design whereby all participants were exposed to a series of four controlled video stimuli varying by emotional valence (distress vs. neutral) and social familiarity (familiar vs. unfamiliar models). Thermal and behavioural responses were measured within subjects across conditions, allowing comparison of emotional reactivity to different social and emotional contexts.

Experimental stimuli were created with two variables of interest: stimulus valence (distress/neutral) and stimulus familiarity (caregiver/stranger). There were four conditions: distress-familiar, distress-unfamiliar, neutral-familiar, and neutral-unfamiliar.

**Fig 1:**
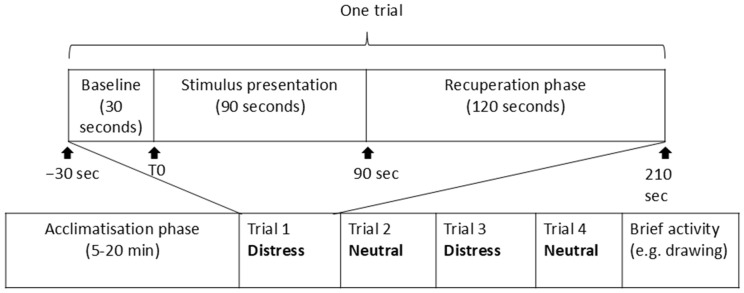
Structure of an experimental session. This figure illustrates a single experimental session, including the acclimatisation phase, trial order, and post-test activity. It also shows the three phases of a trial (i.e., baseline, stimulus presentation, and recuperation), with durations.

Each participant engaged in all four conditions in a single session (25–30 minutes in duration), with a single trial for each condition. A trial was composed of three phases: baseline (30 seconds), stimulus presentation (90 seconds), and a recuperation phase (120 seconds) (Fig 1). Thus, each trial lasted 4 minutes.

The presentation of conditions by valence was of fixed order, distress-neutral-distress-neutral, in order to limit the emotional impact potentially triggered by two successive distress conditions. As exposure to distress scenes could be over-arousing for children, we always ended the session with a neutral condition. If the first trial was with a familiar caregiver, the second trial contained the corresponding neutral condition with the familiar caregiver and the third and fourth trials contained conditions with the unfamiliar caregiver, and vice versa. From Nursery A, 14 out of 21 children (66.7%) started the trial with the videos of their familiar caregivers, while in Nursery B this was 5 out of 9 children (55.6%) (see S2 Table in S1 File). The slight unbalance for Nursery A is due to the exclusion a posteriori of some trials (see *Participants*). While we attempted to complete all trials in a single session for each child, if the session was interrupted, the child was tested on a subsequent occasion for the missing condition(s) only.

### Experimental stimuli

**Fig 2:**
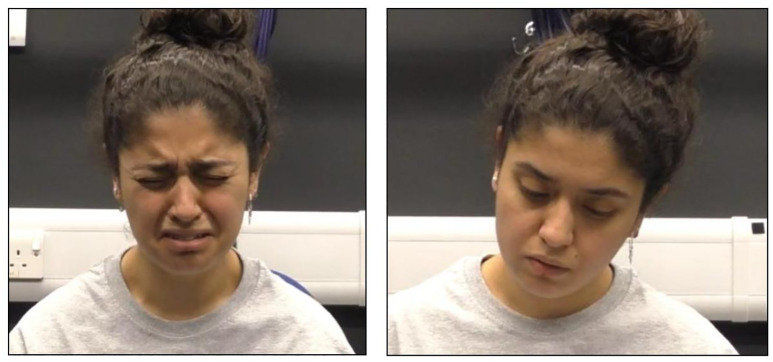
Examples of images from the experimental stimuli. Screenshot exemplars show the distress stimuli (left) and neutral stimuli (right), with permission.

We selected three Nursery caregivers (all female, aged approx. 25–55 years) who consented to participating to create video stimuli. We asked caregivers to simulate an emotional response of either distress or neutral valence. For the *distress condition*, they were provided with training and asked to express sadness (facial expressions of sadness, hunched body movements, sobbing vocalisations) for 1 minute 20 seconds before visibly showing themselves to feel better for 10 seconds and going back to neutrally reading a leaflet placed on their lap, concluding the distress episode and mirroring the control condition. This final component was to ensure the vignette ended on a positive note for the child. See the video example in the Supplementary Material (S1 Video) and Fig 2. No specific reason for their distress was shown or depicted.

In the *neutral condition*, the caregiver was asked to look to be reading a leaflet neutrally placed in their lap while emitting neutral vocalisations (such as ‘hmmm’) to match the presence of audio in the distress condition. Before recording, we showed each caregiver an exemplar video upon which to model performance and pointed out the crucial criteria (e.g., movements, vocalisations) to ensure consistency. The realism and intensity of all of the video clips recorded was evaluated by D. A. and an independent coder, and only realistic videos were retained. We excluded, for example, breaking character (e.g., laughing during the distress display) or not showing the emotion required, or if the emotion shown was too intense (e.g., very loud vocalisations and intense facial expressions) or not intense enough (e.g., no change in facial expression, few vocalisations). There was perfect agreement between the coders as to this quality check. See the video example in the Supplementary Material (S2 Video).

To manipulate familiarity and to control for various visual components, the caregivers from Nursery A were used as the ‘familiar’ individuals to the children in their nursery, whereas they were used as the ‘unfamiliar’ individuals to the children in Nursery B, and vice versa.

### Equipment

We used an infrared thermal camera (T530, FLIR, Stockholm, Sweden; temperature sensitivity <40 mK, IR resolution of 320 x 240 pixels, accuracy (drift): ± 0.3°C) to record the face of the participants with a 29-mm Lens Field of View (FOV, 14° x 10° - FLIR T199588 Tele 14° Lens, auto-emissivity correction 0.01 to 1.0). The IRT camera was on a tripod 1 to 1.3 metres from the child and aimed at their face. A high-definition camcorder (Panasonic HC-V770) recorded the child’s behaviour. We displayed the video stimuli on a 13-inch screen from a Dell laptop (maximum brightness and volume 60) placed 1 to 1.3 metres from the subject at eye height.

Participants’ thermal responses can be sensitive to fluctuations in ambient temperature and humidity. To control for this, we monitored the temperature and humidity of the place/room at least every minute throughout the experiment, with an LCD digital psychrometer temperature and humidity meter (Preciva, Resolution 0.01% RH, 0.01 °C/0.01 °F; see the S1 File for details). This was entered into separate analyses.

### Procedure

Each child was tested individually in a quiet room within their nursery setting. During the session, they viewed a series of short video vignettes featuring both familiar (their primary caregiver) and unfamiliar adult models displaying either distress or neutral expressions. To enhance the ecological validity of the familiar caregiver condition, the child’s own key caregiver was not physically present in the room during the experiment. Instead, another familiar staff member, not featured in any of the video stimuli, remained with the child to provide emotional security and support the child’s well-being. This arrangement minimised potential confounds while supporting a comfortable and ethically appropriate testing environment.

As IRT is sensitive to ambient conditions and bodily movement, careful attention was paid to monitoring this. Test rooms were kept at a generally constant temperature and humidity, with no direct sunlight or ventilation. Within testing sessions, the temperature varied between 0.01 and 0.28 Celsius degrees (SD value, 0.13 Celsius degrees), and the humidity varied between 0.14% and 2.49% (SD, 0.54%). Before testing, participants were in another room of similar temperature and humidity to the testing room. If children were playing outdoors prior to research sessions, we waited at least 15 minutes before starting the trial to allow for acclimatisation. As a caregiver was present, all doors stayed closed during the experiment to prevent airflow and temperature change. The accompanying nursery caregiver was asked to limit their movements and avoid eye contact with the participant.

Because a changing environment can affect nasal temperature [20], there was an acclimatisation phase of at least 5 mins at the beginning of a session to allow the child to adapt to the temperature and humidity of the room [19,20]. During the acclimatisation phase, the child played quietly (colouring, looking at books, playing with blocks) until they showed no behavioural sign of excitement or anxiety for around 5 minutes*.*

**Fig 3:**
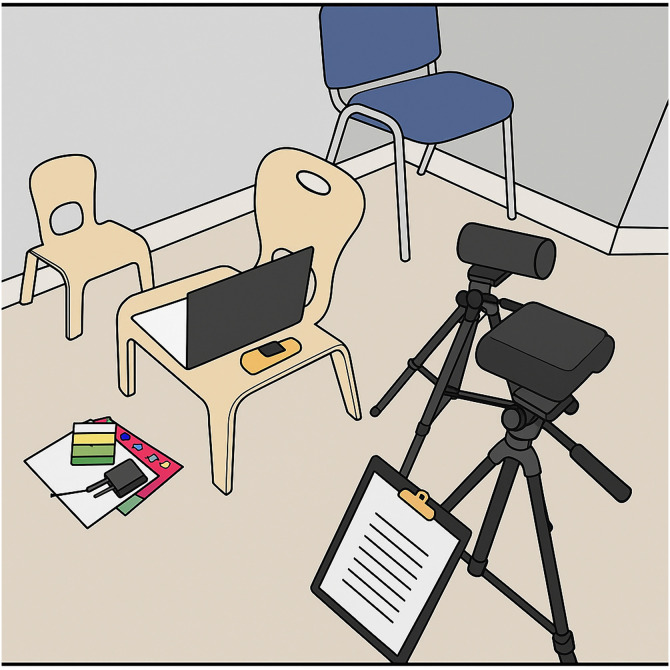
Experimental set-up. Shown are the thermal camera (lower right), video camera (middle right), laptop, the temperature and humidity logger (behind the laptop), stop watch, and chairs for the child and caregiver*.* The thermal camera was positioned after the child was seated at a distance of 1-1.3 metres. This figure was reproduced from a photograph of the real setup using AI.

As movement during the trial can impact thermal measurement [30], we reduced movement by securing participants into a familiar children’s chair. The youngest children sat on their caregiver’s lap. If the child was sitting in the children’s chair, their nursery caregiver sat 1–2 metres away, avoiding visual or physical contact (Fig 3). The child was free to seek contact with the experimenter or the caregiver who was present in the room, but the caregiver was instructed to not respond.

A trial started with a *baseline phase*: a 30-second silent video of bubbles, aimed at measuring the basal/initial facial temperature of the child and behaviour before each of the experimental conditions. We selected the bubbles to attract the attention of children while not being overly arousing.

After this followed an experimental stimulus lasting 90 seconds.

Immediately after the stimulus phase was the *recuperation phase* [53], composed of 120 seconds of neutral silent clips displaying colourful moving shapes on the screen (Microsoft Windows© screensavers). The duration of the recuperation phase (2 minutes) balanced the delay necessary for the child to return to baseline following the potentially arousing stimuli and the child’s attention span [54,19].

During the session, the experimenter and caregiver closely monitored participant responses in order to cease the experiment if any participant showed excessive distress; however, this did not occur. The test session concluded with an enjoyable activity, such as drawing, playing, or reading a book to ensure the session ended on a rewarding note.

### Coding

#### Coding of Infra-red thermal data.

**Fig 4:**
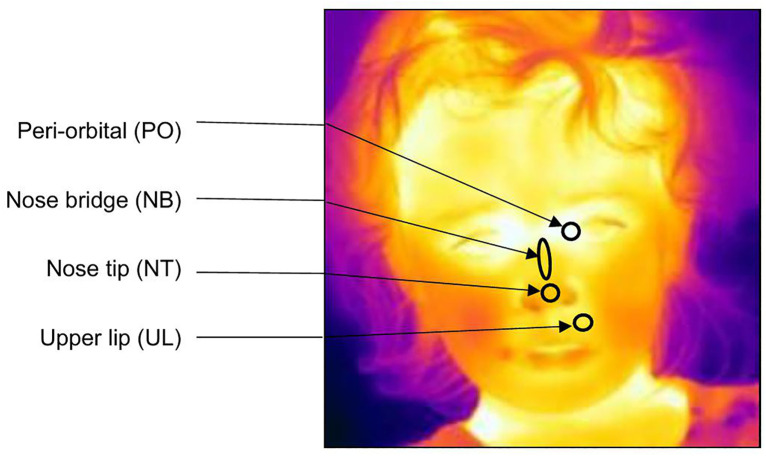
The four facial regions of interest (ROIs). The name and shape of each ROI, as well as its location on the face, are shown.

We used FLIR Tools software to process the thermal videos. We first inspected the thermal profiles for a subset of the data to establish the precise coding protocol (N = 15 children – see the Supplementary Material S3 Video); we did so by extracting minimum temperatures every 10 seconds during baseline (i.e., 10-second intervals from −30–0 seconds relative to the stimulus onset), stimulus presentation (i.e., from 10 to 90 seconds relative to stimulus onset), and recuperation (i.e., from 10 to 120 seconds relative to stimulus offset) phases at the four regions of interest (ROI), i.e., periorbital (circular shape), nose bridge (ellipsoidal shape), nose tip (circular shape), and upper lip (circulate shape) (Fig 4). ROIs were manually placed on each frame using thermal imaging software, with the shapes selected to match the anatomical contours and to optimise consistency across participants and timepoints. The ellipsoidal shape for the nose bridge allowed for the elongated structure of the area, while the circular ROIs were suited for smaller, more discrete regions such as the tip of the nose and periorbital zones. Ultimately, only the first three ROIs were retained (see Results). Raw thermal data (minimum, maximum, and average temperature within each ROI extracted every 10 seconds) were visually inspected.

For the full data set, we extracted the IRT temperature at three time points. (1) The ***baseline temperature*** was taken at −10 seconds relative to stimulus onset. (2) The **stimulus offset** temperature was taken near the end of the stimulus display (+80 seconds from stimulus onset). This was chosen because changes in facial skin temperatures are known to be relatively slow compared to other responses of the autonomic nervous system [20,55]. (3) The **recuperation phase** time point was near the end of the recuperation phase (+200 seconds from stimulus onset), to capture a potential return to baseline. Following Kano and colleagues [20], we extracted the minimum temperature inside each ROI for precision. Frames were selected within ±3 seconds of the target frame (e.g., for the 80-second time point, frames between 77 and 83 seconds of stimulus onset could be used) for each of the four trials. This allowed for selection of a high-quality frame [19], and avoidance of blurring/head motion [20]. When we could not find an optimal frame within ±3 seconds, measurement time-points were coded as null values.

#### Coding of the behavioural data.

We used ELAN, an open-source annotation software for audio and video recordings (Version 5.9, Nijmegen: Max Planck Institute for Psycholinguistics, The Language Archive), to code the behavioural responses of children during the experiment [e.g., 56].

**Fig 5:**
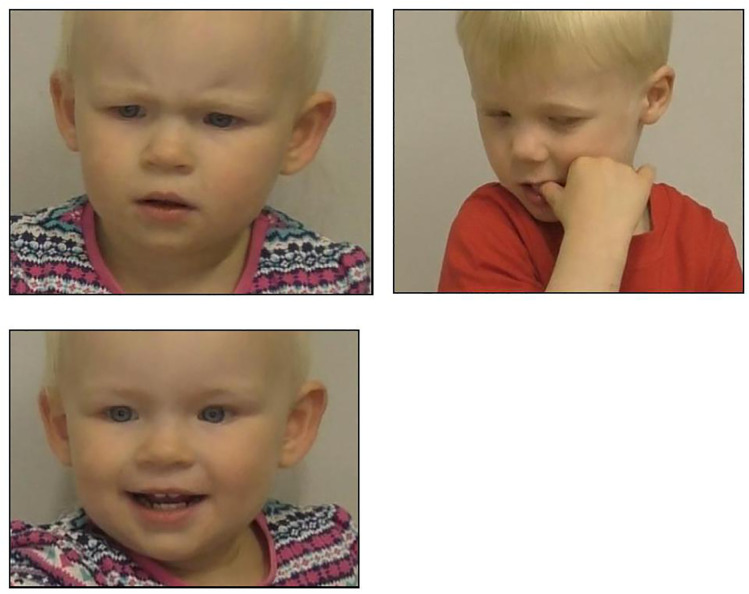
Facial expressions of some participants. Concern is shown by a girl in the unfamiliar distress condition (e.g., brow furrowed, open mouth) (top left panel); discomfort is shown by a boy in the familiar distress condition (e.g., avoiding look, hand in mouth) (top right panel); and amusement and happiness by a girl in the familiar distress condition (i.e., mouth smiling and cheeks raised) (bottom left panel). All photos are included with parental permission.

Using a detailed ethogram, we coded the following behavioural responses: (1) duration and occurrence of *looking/attention* towards four sources: the screen, the caregiver in the room, the experimenter, or looking away from the screen or either adult; (2) the duration and occurrence of *facial expressions* (i.e., concern and discomfort, along with other negative facial expressions, including distress; positive facial expressions; neutral facial expressions; and others); (3) the duration and occurrence of *self-directed behaviours* (e.g., self-scratching); and (4) the duration and occurrences of *pointing* behaviours towards the screen. Behaviours within each category are mutually exclusive. See S1 File for the ethogram and Fig 5 for examples.

#### Duration and occurrence of looking/attention.

Participant attention towards the stimulus was recorded when their face was visibly facing the screen with an angle of ±20 degrees for at least one second. This threshold was selected based on established conventions in developmental research, which indicate that attention and gaze direction can be reliably inferred from small head movements in this age group. The 20-degree range allowed for natural minor head movements while still reflecting sustained engagement with the screen content.

Looking away was coded when their face was outside of the 20-degree angle relative to the screen, and/or the participant’s gaze indicated that the child was not looking at the screen for at least 1 second, indicating disengagement. Participant was coded as “looking at the caregiver” when the face of the child was oriented towards the caregiver (± 20-degree angle) for at least 1 second. This involves a more distinct rotation of the head threshold of more than a 35-degree angle from the screen after looking at the stimulus, suggesting that child sought visual comfort/support and/or social referencing (i.e., contingency of looking at the stimuli and then looking at the caregiver in the room). The participant was coded as “looking at the experimenter” following the same criteria. These criteria aligned with the physical layout of the room, in which the caregiver and experimenter were positioned to the side and behind the child’s line of sight to the screen, requiring a deliberate head turn of ~35 degrees or more to make visual contact. This setup ensured that coded instances of looking towards the caregiver or experimenter reflected purposeful social engagement rather than incidental gaze shifts.

#### Facial expressions.

Coding of the facial expressions of concern, discomfort, and distress was conducted in two phases. We first coded the occurrence of any *negative facial expressions*, which included any signs of concern, discomfort, and distress (disgust, sadness, fear, and anger were not observed). From these negative facial expressions, we then specifically identified concern, distress, or discomfort, which were all mutually exclusive (Fig 5).

To further verify that children accurately perceived the distress condition as negative, we also coded participants’ positive facial expressions, predicting positive expressions to occur more often in the neutral than distress condition. This included coding expressions of amusement, happiness, or excitement, e.g., mouth smiling and cheeks raised [57].

#### Duration and occurrence of self-directed behaviours and pointing.

**Fig 6:**
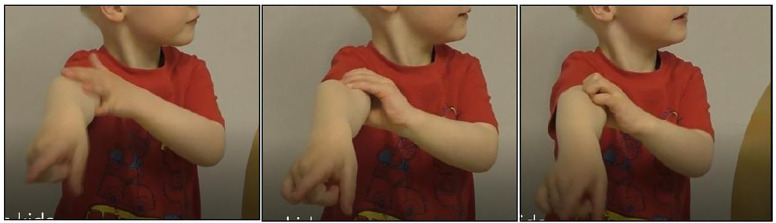
Self-directed behaviours and pointing. Examples of self-directed behaviours and simultaneous pointing (all three panels) shown by a boy in the familiar distress condition – with permission.

Self-directed behaviours were defined as when the participant made soft repeated scratching movements or a sharp single movement with their fingertips (with or without the use of nails) on their own body, as coded in the non-human primate literature [36; 37]. It also included the manipulation of one’s hair, clothing, and/or accessories (Fig 6).

When the child raised and extended their arm, hand, and at least one finger towards the screen, it was coded as “pointing behaviour” (Fig 6).

### Statistical analysis

#### Inter-coder reliability.

15% of the total thermal and behavioural data was second coded by two research assistants unaware of the study condition. See S1 File for details of how this analysis was performed.

#### Analyses.

For thermal analyses, we used the minimum temperature at each ROI. We measured the temperature change from 80 and 200 seconds after stimulus onset compared to baseline. We inspected the distribution of the covariate and the distribution of the response. No data transformation was conducted because they were near normal distribution.

For behavioural analyses, we used ELAN to code the duration of each facial expression and behaviour within the 30 seconds following stimulus onset as compared to the 30-second baseline. Data were close to the normal distribution, centred around zero.

For the attention/looking measure, durations when the participant looked at the caregiver in the room or the experimenter were excluded.

#### Statistical analyses.

We used generalised linear mixed models [GLMMs, 58] using the functions lmer and glmer (version 3.1–1) of the package lme4 [version 1.1–25; 59], along with the optimizer ‘bobyqa’ to improve model fit, in R [version 3.6.3; R Core 60]. The response variables, fixed effects, and control variables are detailed for each model in Table 1.

**Table 1:** Variables entered into the respective models.

*Variable*	*Level*
***Fixed effects***	
*Stimulus Familiarity*	*Familiar; Unfamiliar*
*Stimulus Valence*	*Distress; Neutral*
*Participant Sex*	*Male; Female*
*Participant Age*	*Continuous (in months)*
***Control variables***	
*Trial order*	*1; 2; 3; 4*
*Stimulus ID*	*3 combinations of 2 familiar/unfamiliar caregivers*
*Nursery*	*Nursery A; Nursery B*
***Random slope***	
*Familiarity + Valence | ID*	
***Random Effect***	
*Participant ID*	
***Interaction***	
Familiarity * Valence	

To detect developmental and sex-based differences, age in months and participant sex were included in the models. Control variables include trial order (1–4), stimulus ID (i.e., caregiver identity), and nursery (A/B). To control for the potential interaction between random and fixed effects, participant ID nested within stimulus familiarity and within stimulus valence were included as random slopes (Table 1).

We examined whether changes in the following physiological and behavioural measures were predicted by stimulus valence (i.e., distress vs neutral) and model familiarity (familiar vs unfamiliar): (i) change in facial skin temperature relative to baseline (relative change at 80 and 200 seconds following stimulus onset for each ROI; continuous), (ii) duration of avoidance behaviours (i.e., looking away; continuous), (iii) duration of concern facial expressions (continuous) and presence/absence of concern facial expression (binomial), (iv) duration of other negative facial expressions (continuous), (v) duration of positive facial expressions (continuous), (vi) occurrence of self-directed behaviours (count), and (vii) pointing behaviours (count). For the continuous response variables, models were Gaussian with identity link functions; for the binomial response variable, the model was binomial with a logit link function.

Overall, we ran 11 full GLMMs. Full models were all first compared to null models, which contained only the intercept, control variables, random effect, and random slopes.

We also conducted additional analyses to account for environmental effects of humidity and room temperature, please see S1 File.

#### Data access.

All data have been made publicly available and can be accessed in S2 File (and here). This study was not pre-registered.

## Results

Each child (N = 30) completed 4 trials, resulting in a total of 120 trials.

### Inter-coder reliability

For thermal analyses, there was an excellent absolute agreement between the three coders for the nose bridge (kappa = 0.994, *p* < 0.01) and the nose tip (kappa = 0.998, *p* < 0.001) regions, and a moderate [61] absolute agreement for peri-orbital (kappa = 0.677, *p* < 0.001) and upper lip (kappa = 0.643, *p* < 0.001) regions (see S3 Table in S1 File). Given its lower reliability, the u*p*per lip region was excluded from analysis.

For behavioural analyses, the Cohen’s kappa was good to excellent for duration and occurrences: including attention towards the emotional display (kappa = 0.91) and facial expressions (kappa = 0.85). The facial expression of concern was coded as a subset of the negative facial expressions.

### Infra-red thermal imagery

#### Nose bridge.

**Fig 7:**
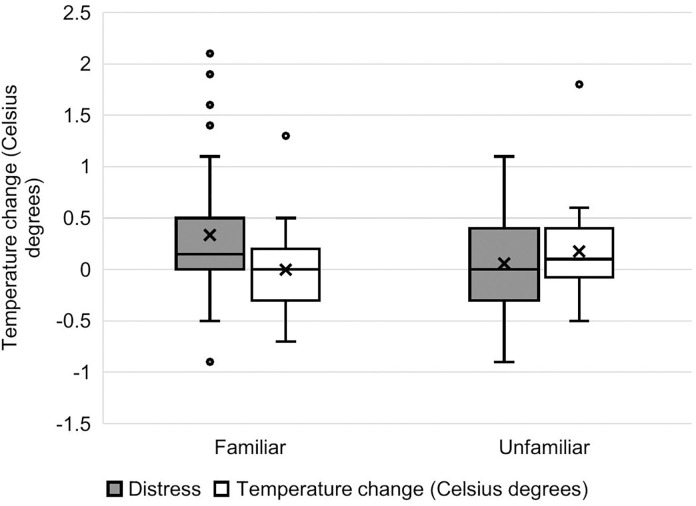
Nose bridge temperature change from baseline 80 seconds after stimulus onset. Temperature change is shown as a function of familiarity of the model and condition (distress or neutral). The bars across the boxes are medians; crosses represent means. Dots represent outlier data that are outside 1.5x the interquartile range (IQR), up and down.

The model of temperature change 80 s after stimulus onset just failed to reach significance over the null model (*χ*^2^ = 9.73, d.f. = 5, *p* = 0.083); therefore, results should be treated with caution. There was a significant interaction between familiarity and valence on nose bridge temperature: compared to baseline, nasal temperature increased more strongly in the familiar-distress condition compared to the familiar-neutral condition, while it increased more in the unfamiliar-neutral condition compared to the unfamiliar-distress condition (estimate ± se = 0.46 ± 0.17, t = 2.81, *p* = 0.004). There was no effect of participant age or sex (S5 Table in S1 File and Fig 7).

**Fig 8:**
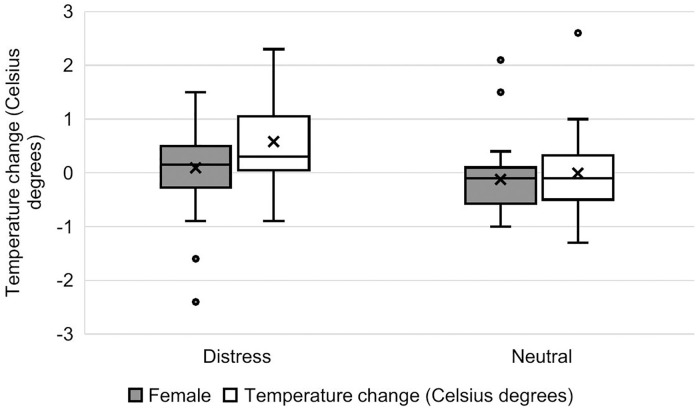
Nose bridge temperature change from baseline at 200 seconds after stimulus onset. Change in temperature is shown as a function of sex of the participant and condition (distress and neutral). Bars across the boxes are medians; crosses represent means. Dots represent outlier data that are outside the 1.5x the IQR, up and down*.*

The model examining recuperation (200 s after stimulus onset) for the nose bridge was significantly better than the null model (*χ*^2^ = 12.59, d.f. = 5, *p* = 0.027). The Familiarity*Valence interaction was not significant, therefore we removed it from the model and re-ran it without this interaction. There was a significant main effect of valence but not of familiarity on nose bridge temperature after recuperation.Compared to baseline, nose bridge temperature at 200s was more elevated in the distress condition than the neutral condition (estimate ± se = −0.58 ± 0.23, t = −2.56, *p* = 0.012; S6 Table in S1 File and Fig 8). There was also an effect of sex, with nose bridge temperature being relatively higher at recuperation in boys than girls, regardless of valence or familiarity (estimate ± se = 0.38 ± 0.17, t = 2.21, *p* = 0.024) (Fig 8). There was no effect of participant age on temperature change.

#### Nose tip.

For the nose tip region at 80s after stimulus onset, the full model failed to reach significance over the null model (*χ*^2^ = 8.056, d.f. = 5, *p* = 0.153).

**Fig 9:**
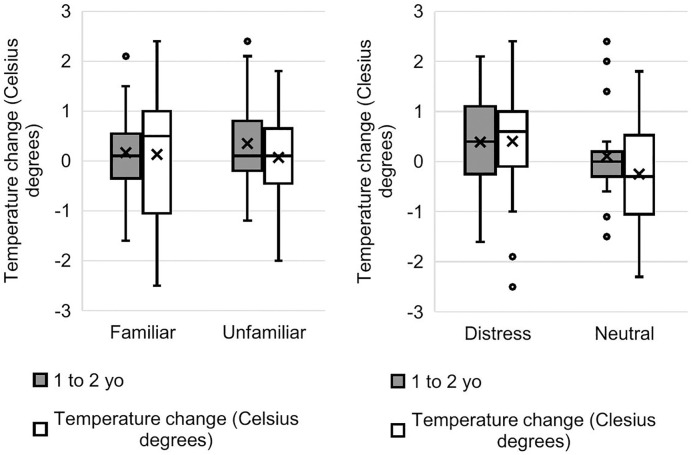
Nose tip temperature change from baseline at 200 seconds after stimulus onset. Temperature change is shown as a function of the age (yo = years old) of the participant and familiarity of the model (left) and as a function of the age of the participant and valence of the stimulus (right). Bars across the boxes are medians; crosses represent means. Dots represent outlier data that are outside the 1.5 times the IQR, up and down.

The full model for recuperation phase (200s) for the nose tip explained the data marginally significantly better than the null model (*χ*^2^ = 10.04, d.f. = 4, *p* = 0.040). The Familiarity*Valence interaction was not significant, therefore it was removed from the model. There was an effect of age: nose tip temperature increased more strongly in younger children than older children (estimate ± se = −0.83 ± 0.42, t = −1.98, *p* = 0.039; S7 Table in S1 File and Fig 9).

The following effects did not reach significance at the *p* < 0.05 level; we discuss them as interesting phenomena for potential future exploration. There was a marginal sex effect, with nose tip temperature at 200s increasing more in boys than girls (estimate ± se = 0.38 ± 0.21, t = 1.87, *p* = 0.057). There was also a marginal effect of valence but not familiarity at the recuperation phase relative to baseline: nose tip temperature tended to increase more strongly in the distress condition than the neutral condition (estimate ± se = −0.45 ± 0.24, t = −1.86, *p* = 0.063; Fig 9, right panel).

#### Peri-orbital area.

For the peri-orbital region at both 80s and 200s after stimulus onset, the full models failed to reach significance over the null models (for 80s: *χ*^2^ = 1.98, d.f. = 5, *p* = 0.852; for 200s: *χ*^2^ = 4.70, d.f. = 5, *p* = 0.454).

#### Temperature and humidity.

The full-null model comparisons did not reveal any effect of the room temperature and humidity on the thermal response in the three ROIs at 80 or 200 seconds after stimulus onset (*p* > 0.05).

### Behavioural analyses

Full-null model comparisons were not significant for avoidance (looking behaviour), concern facial expressions, other negative facial expressions, self-directed behaviours, or pointing (S8 Table in S1 File). Over-dispersion was no issue in any model, and no considerable collinearity was detected following inspection of variance inflation factors.

As these were secondary to the main study goals, results for positive expressions are provided in the supplementary results (see S9 Table and S1 Fig in S1 File).

## Discussion

In this study, we investigated the development of emotional responding to others’ distress through a multicomponent approach. We measured children’s emotional responses to emotional and neutral video stimuli, using a combination of non-invasive infra-red thermal imagery and corresponding analysis of facial expressions and behaviours.

Overall, the thermal imagery findings indicate an emotion contagion reaction of children (at the nose bridge at 80s) in response to others’ distress. Moreover, in line with the empathy gradient (Preston & de Waal, 2002)*,* children’s facial temperature at the nose bridge area increased more strongly when observing familiar individuals in distress as compared to unfamiliar. An elevated temperature at the nose bridge during the recovery phase (200s) was predicted by stimulus valence and sex, and at the nose tip by younger age.

As predicted according to the principle of emotion contagion, there were stronger skin temperature changes in response to the distress condition compared to the neutral condition. In this case, the direction of change was positive, which contrasts with a previous developmental study in which the nasal skin temperature decreased in children and their mothers in a distressing situation [54]. Although the mechanisms shaping the directionality of thermal responses are not yet well understood, it is possible that personally experiencing versus observing distress may affect the nature of the thermal response. In the study of Ebisch et al. [54], the child directly experienced the distressing situation (by breaking what they thought was the researcher’s favourite toy), rather than vicariously witnessing another’s distress. The non-human primate literature also indicates a decrease of skin temperature in the nasal area (usually nose tip) in response to experiencing negative situations like fear [20,55,62] or in association with the vocalisations of conspecifics [22]. Previous human studies highlighted mixed results for temperature changes, which can vary according to stimulus valence as well as the region of interest used [decreases: 63,30,64; increases: 65,64,14,66]. Overall, given these equivocal results, further work with larger sample sizes in controlled conditions is needed to investigate the nature of the underlying arousal responses that thermal-imaging is measuring.

Mechanistically, increased skin temperature in certain facial regions might result from vasodilatation leading to increased blood flow connected to an increase of the heart rate along with α- and β-adrenergic influences [67]. For instance, responding to danger activates a *flight* or *fight* response [68] which may induce increased heart rate and shallow breathing, while at the same time diverting energy from other parts of the body, including the digestive system as well as also other parts of the face, due to vaso-constriction [68]. Such reactions could explain why one can observe both increases and decreases in facial temperature at different regions of interest [69].

Our thermal data also revealed some developmental variation, with the strength of the thermal response (in the nose tip region at 200s) decreasing with age. This supports the possibility that through development, children may gain control of the inner arousal response via their developing emotion regulation skills, or at least be less emotionally aroused by their surrounding environments [70,71]. One alternative explanation for the age effect is that the older children cared less about the situation and were therefore less emotionally affected by it. Another is that as children grow, their facial dermal properties change (e.g., thicken), which blunts their thermal responding.

Hypothesised differences in children’s behavioural responses to the stimuli based on valence, sex, age, or familiarity were not found. However, there was a marginally significant effect of valence on positive facial expressions, with more positive expression in the neutral condition compared to the distress condition. In this respect, despite evidence of a thermal signature, our behavioural data did not support evidence of either emotional contagion or empathic concern. The latter would be evidenced by greater facial expressions of concern towards the distress stimuli, which we did not find. This suggests that thermal imagery may detect more subtle physiological responses to others’ distress that are not necessarily visible within the behavioural dimension, highlighting the need for more multi-componential studies. Methodologically however, behavioural and thermal responses occur on different time scales, with thermal responses relatively slow to occur [55], whereas behavioural responses are typically rapid. In this respect, as physiological and behavioural responses might reflect different processes, their profiles may not be directly related.

What might explain the lack of a predicted difference in behavioural responses, in the form of self-directed behaviours and facial expressions of concern, between the emotional (distress) and neutral stimuli? Several possibilities present themselves, relating to both aspects of the stimuli and the experimental procedure. Previous experimental studies have used visual contextual cues, like pretend injuries [6,72,73] and need for help [74,75], to elicit emotional responses in children. However, the outputs of these studies are somewhat hard to interpret, as it is unclear whether the children were reacting to the emotion of the individual or to the distressing situation itself. In the present study, the models simulated distress with no contextual cues as to what caused the distress. Although this controlled for potential confounds, it also reduced ecological validity by using context-free events, which were posed rather than spontaneous. The interpretation of experiments using posed stimuli is challenging [76,77]. Previous studies have presented subjects with posed demonstrations [children: 72; 78], video stimuli [79,80,81], or genuine audio stimuli [i.e., playback experiments, 72,8]. Despite evidence that actors often amplify stereotypical displays of emotions [82], there is still evidence that such stimuli can constitute a good resemblance to genuine emotional displays [83,84]. Previous attempts to use still images or video footage failed at eliciting physiological reactions in chimpanzees [85], potentially because the visual stimulus was too weak. Although the stimuli used in the present study elicited a thermal response in the child participants, it is possible that the combination of being posed and being context-free inhibited the expected behavioural responses. Further work should address this issue of ecological validity in experimental designs of emotional responding.

For ethical reasons, it was necessary to have a nursery caregiver in the room during the experiment. Nevertheless, the presence of others influences the behaviour of an individual through social facilitation and audience effects [86,87,88]. It is possible the presence of two adults in the audience inhibited children’s behavioural responses, especially if children perceived them as authority figures [89,19]. Future work might carefully consider the impact that social audiences have on children’s physiological and behavioural responding.

In contrast to our predictions, we found no age effects for the behavioural measures. The age range of the sample extended from 1 to 3 years, a period of rapid socio-cognitive and communicative development and physical mobility. With increased socio-cognitive skills, we expected to see a shift in the coping strategies employed by children in the different situations. However, it is possible that the use of acted stimuli, as well as the presence of the caregiver and the experimenter in the room [*audience effect,* 88*; social facilitation*, 86,90,91,87], might have reduced children’s behavioural expressions. Future work using more naturalistic scenarios and stimuli could expand the investigation. This could even expand to examine the use of different facial expressions as indicators of affective states, but also emotion regulation strategies, such as to elicit social support and lower physiological arousal [92,93,94].

Although there was a sex difference in recuperation phase (200s) nose-bridge temperature (with boys’ temperatures more elevated than girls’), predicted behavioural sex differences were not apparent, in either direction. This could be due to some of the factors listed above.

## Conclusion

This mixed-methods study confirmed a physiological reaction of children when witnessing another’s distress. Although more clearly an indicator of emotion contagion, evidence of a social bias towards familiar individuals in distress supports the proposed social gradient of empathy. Taken together, our results did not reveal a clear link between behavioural and physiological responses of children to the stimuli, with evidence of thermal responses in the absence of corresponding behavioural expressions. Our study contributes to new theoretical and methodological advances into how internal affective processes map onto external measures in early childhood. It also highlights the need to combine multiple measures, including IRT, but also other noninvasive measures such as heart rate, pupil dilation, and behavioural markers, to improve our understanding of the underlying affective mechanisms. Overall, the results of this study highlight the complexity of measuring emotional responding.

## Supporting information

S1 File Supporting information. This file contains S1 to S9 Tables, S1 Fig., the ethogram, additional information about the method, and references.
(DOCX)

S2 File Thermal data. This file contains anonymised data for all participants.
(XLSX)

S1 Video Distress Stimuli example. (MP4)

S2 Video Neutral Stimuli example. (MP4)

S3 Video Example of infra-red thermal imaging video. (MP4)
