# Supplementary material for: How do young children respond to the distress of others? Applying infrared thermography and behavioural analyses to examine the development of emotion contagion and empathy
Source: PLoS One. 2026 Mar 4;21(3):e0335537. doi: 10.1371/journal.pone.0335537 (PMC12959689; doi:10.1371/journal.pone.0335537)
Supplement: S1 File — This file contains S1 to S9 Tables, S1 Fig., the ethogram, additional information about the method, and references. (DOCX) [file pone.0335537.s001.docx]

**How do young children respond to the distress of others? Applying infrared thermography and behavioural analyses to examine the development of emotion contagion and empathy**

**Supplementary material**

**Demographic Information**

To participate in the study, children must have joined the Nursery at least 8 weeks before participating, and must have attended the Nursery from three to five days a week over the course of these 8 weeks to ensure they had enough time to develop their familiarity with their caregiver.

The individual/participants who feature within the Figure images and the Supporting Information – Video S1, S2 and S3 in this manuscript have provided written informed consent (as outlined in PLOS consent form) to publish these excerpt image details alongside the manuscript.

*Supplementary Table S1* Information about the children included in the IRT and behavioural data sets

|  | **Girls** | | **Boys** | |
| --- | --- | --- | --- | --- |
|  | 1 to 2-years-old | 2 to 3-years-old | 1 to 2-years-old | 2 to 3-years-old |
| Nursery A | 6 | 3 | 6 | 6 |
| Nursery B | 4 | 4 | 1 | 0 |
| **Total** | **10** | **7** | **7** | **6** |

*Supplementary Table S2* Number of children who started the trial with familiar vs unfamiliar condition

|  | **Nursery A** | | **Nursery B** | |
| --- | --- | --- | --- | --- |
|  | *>1 to 2-year-old* | *>2 to 3-year-old* | *>1 to 2-year-old* | *>2 to 3-year-old* |
| Familiar | 9 | 5 | 3 | 2 |
| Unfamiliar | 3 | 4 | 2 | 2 |

**Specifications of the Temperature and Humidity Meter**

Preciva LCD Digital Psychrometer Mini Temperature and Humidity Meter with Dew Point and Wet Bulb Temperature Hygrometer - Specifications

- Display: Large 4-1/2 dual digital LCD display
- Sensor Type: A single chip relative humidity and temperature muti sensor module comprising a calibrated digital output
- Response Time: %RH: 10S (90% at +25 °C still air)
- Accuracy Note: Accuracy is specified for the following ambient temperature range: 64°F to 82°F (18°C to 28°C)
- Sampling Rate: 2.5 samples per second
- Polarity: Automatic, (-) negative polarity indication
- Dewpoint Temperature: -20 °C~80 °C (-4 °F~176 °F)
- Wet Bulb Temperature: 0 °C~80 °C (32 °F~176 °F)
- Operating Conditions: 32°F to 104°F (0°C to 40°C); < 80% RH non-condensing
- Storage Conditions: 14°F to 140°F (-10°C to 60°C); < 80% RH non-condensing
- Power: One standard 9V, NEDA 1604 or 6F22 battery
- Dimensions: 175 x 58 x 35 mm
- Weight: 143g
- Humidity/ Temperature Measurement Range:
  - Humidity: 0%~100%RH
  - Temperature: -20 °C~80 °C (-4 °F~176 °F)
  - Resolution: 0.01%RH, 0.01°C/0.01°F
  - Humidity Accuracy: ±3%RH (at 25°C, 20%~80% RH); ±3.5%RH (At other ranges)
  - Air Temperature Accuracy: ±0.5°C/±0.9°F (at 25°C); 0.8°C/±1.5°F (all other ranges)

**The coding system**

The coding system was inspired by the Maximally Discriminative Facial Movement coding system (MAX; Izard, 1979) and some physical criteria identified and described in the empathy coding scheme from the MacArthur Longitudinal Twin Study developed by Zahn-Waxler and Robinson and their colleagues (Zahn-Waxler et al., 1992; Zahn-Waxler & Robinson, 1995), and used extensively in prior research (e.g., Knafo et al., 2008; Davidov et al., 2020).

#### Inter-coder reliability

15% of the total data was second coded by two research assistants unaware of the study condition.

Thermal values were then compared using intraclass correlation coefficients (ICC, Shrout & Fleiss, 1979; Koo & Li, 2016). We used the function icc() from the irr package in R to assess the absolute agreement between the main coder and two sub-coders in extracting the facial temperature from the four regions of interest using the two-way random effect models and "single rater" unit.

Inter-coder reliability of each behaviour in each of three categories (i.e., looking/attention, facial expressions and self-directed behaviours) was calculated between two second coders using Cohen’s kappa on a minimum of 15% of the data set (i.e., 5 children). The coders were unaware of the condition (i.e., distress or neutral, familiar or unfamiliar). To limit inferences between categories, each category was coded separately.

*Supplementary Table S3* Intraclass correlations coefficients (ICC) for absolute agreement of three coders using the two-way random effect models

| Average measures for the regions of interest | Intraclass Correlation | 95% Confidence Interval | | F Test with True Value | | | |  |
| --- | --- | --- | --- | --- | --- | --- | --- | --- |
|  |  | *Lower bound* | *Upper bound* | *Value* | *df1* | *df2* | *Sig* |  |
| Nose bridge | 0.994 | 0.990 | 0.996 | 163.34 | 59 | 118 | <0.001 |  |
| Nose tip | 0.998 | 0.996 | 0.999 | 653.21 | 59 | 118 | <0.001 |  |
| Peri-orbital | 0.677 | 0.442 | 0.811 | 3.91 | 59 | 118 | <0.001 |  |
| Upper lip | 0.643 | 0.342 | 0.800 | 3.84 | 59 | 118 | <0.001 |  |

**Ethogram for children’s behavioural responses**

**Facial expressions**

To code for the facial expression of concern, the participant’s facial expression included sobering, sad expression with corners of the mouth turned down, or ‘sympathy face’ with brow furrow, or lips tight together (Davidov et al., 2020).

Regarding other negative facial expressions, the child could also show distress facial expressions which were defined as eyes wide and mouth open as signs of wariness or fear or discomfort facial expression.

Other less frequent expressions of negative emotion were sadness (inner corners of brows raised, outer corners lowered, eyes narrowed or squinted, corners of the mouth pulled down and out), fear (brows straight or normal, slightly raised or drawn together, eyelids raised and tense, mouth open and corners straight back) or anger (brows down straight or slanting down toward the centre, brows often drawn together, cheek raised) facial expressions (Izard, 1979).

Participants could also produce expressions of positive affect which include expressions of amusement, happiness or excitement, e.g., mouth smiling and cheeks raised (Dimberg & Thunberg, 2012).

**Self-directed behaviours**

Self-directed behaviours were defined as when the participant made soft repeated scratching movements or a sharp single movement with their fingertips (with or without the use of nails) on their own body, as coded in the non-human primate literature (Castles & Whiten, 1998; Koski et al., 2007). It also included the manipulation of one’s hair, clothing and/or accessories.

**Social referencing and information seeking/sharing**

The participant was coded as “looking at the caregiver” when the face of the child was oriented towards the caregiver (± 20-degree angle) for at least 1 second. This behaviour involves a rotation of the head of more than a 35-degree angle from the screen after looking at the stimulus, suggesting that the child seeks visual comfort/support and/or social referencing (i.e., contingency of looking at the stimuli and then looking at the caregiver in the room).

Similarly, the participant was coded as “looking at the experimenter” following the same criteria. However, we did not analyse the time spent by the child looking at the experimenter because they tended to move around to deal with the equipment. When the child raised and extended their arm, hand and at least one finger towards the screen, it was coded as “pointing behaviour”. During this behaviour, the child could have been looking toward the direction of their arm/hand/finger or not.

**Practical limitations**

From more practical stances, the children who could sit down by themselves were asked to sit on a chair, while the younger ones were on the laps of their caregiver. It is conceivable that both the behavioural and physiological measurements had been impacted by these settings. Indeed, sitting on a chair may constitute a physical barrier to some behavioural responses such as approaching (Abramson et al., 2019). Also, the infants who were on the laps of their caregivers might have been impacted by the body heat of the caregiver, as well as by their movements. Future work should focus on designing experiments that allow for enough control to permit clear thermal measurement, while conveying as naturalistic conditions as possible to enable the children to express themselves with minimum constraints.

**IRT data description**

For the 360 measurements, the ideal frames were extracted on average at ±2.4 seconds (SD = 2.8 seconds; minimum = 0 seconds; maximum = 9 seconds around 80 and 200 seconds after the onset of the stimulus). See below the mean skin temperatures of the three measured facial areas of the children when tested for distress and neutral situations (Table S4).

*Supplementary Table S4* Mean (±SD) skin temperatures (Celsius degrees) of the three measured facial areas of the children when tested for the distress and neutral situations

|  |  | Familiar | | Unfamiliar | |
| --- | --- | --- | --- | --- | --- |
| Time points | Region of interest | *Distress* | *Neutral* | *Distress* | *Neutral* |
| Baseline | *Peri orbital* | 35.0 (±0.7) | 35.0 (±0.7) | 35.0 (±0.6) | 35.1 (±0.7) |
|  | *Nose bridge* | 33.5 (±1.8) | 33.9 (±1.6) | 33.6 (±1.8) | 33.7 (±2.0) |
|  | *Nose tip* | 32.8 (±2.1) | 33.3 (±1.9) | 32.8 (±2.0) | 33.1 (±2.1) |
| 80 sec  (Test) | *Peri orbital* | 35.0 (±0.5) | 35.1 (±0.7) | 35.0 (±0.7) | 35.1 (±0.5) |
|  | *Nose bridge* | 33.8 (±1.7) | 33.9 (±1.7) | 33.6 (±1.9) | 33.8 (±1.9) |
|  | *Nose tip* | 33.3 (±2.1) | 33.3 (±2.0) | 33.0 (±2.1) | 33.3 (±2.0) |
| 200 sec  (Recuperation) | *Peri orbital* | 35.0 (±0.7) | 35.0 (±0.5) | 35.1 (±0.6) | 35.0 (±0.7) |
|  | *Nose bridge* | 34.0 (±1.4) | 33.6 (±1.8) | 33.8 (±2.0) | 33.5 (±1.7) |
|  | *Nose tip* | 33.2 (±1.9) | 33.0 (±2.0) | 33.2 (±2.2) | 33.0 (±2.1) |

**Room temperature and humidity**

For models with thermal measures as response variables, we ran separate models with the temperature (Celsius) and humidity (percentage) variation within a testing session (standard deviation values) as sole fixed effects separately. These models were compared with the null models. No significant changes in temperature and humidity were recorded during the trials.

**Supplementary Results**

*Supplementary Table S5* Results of the full model testing the effect of familiarity, valence, sex, and age on temperature change at the nose bridge region at 80 seconds

|  | Estimate | S.E. | t value | LRT | P |
| --- | --- | --- | --- | --- | --- |
| (Intercept) | 0.290 | 0.422 | 0.689 |  |  |
| Familiarity(Unfamiliar) * Valence(Neutral) | 0.464 | 0.165 | 2.808 | 8.087 | **0.004** |
| Familiarity (Unfamiliar) | -0.059 | 0.095 | -0.622 | 0.430 | 0.512 |
| Valence (Neutral) | -0.139 | 0.124 | -1.120 | 1.312 | 0.252 |
| Age | 0.010 | 0.231 | 0.044 | 0.002 | 0.962 |
| Sex (Male) | 0.049 | 0.113 | 0.435 | 0.204 | 0.651 |

Significant values (P<0.05) are in **bold.** S.E.: standard error; LRT: Likelihood Ratio Test; P: p-value.

*Supplementary Table S6* Results of the full model testing the effects of familiarity, valence, sex, and age on temperature change at the nose bridge at 200 seconds

|  | Estimate | S.E. | t value | LRT | P |
| --- | --- | --- | --- | --- | --- |
| (Intercept) | 0.866 | 0.632 | 1.371 |  |  |
| Familiarity (Unfamiliar)^a^ | -0.203 | 0.207 | -0.985 | 0.262 | 0.609 |
| Valence (Neutral) | -0.581 | 0.227 | -2.556 | 6.370 | **0.012** |
| Age | -0.361 | 0.349 | -1.034 | 1.161 | 0.611 |
| Sex (Male) | 0.375 | 0.170 | 2.205 | 5.110 | **0.024** |

Significant values (P<0.05) are in **bold.** S.E.: standard error; LRT: Likelihood Ratio Test; P: p-value. ^a^, The familiarity-valence interaction term was not significant, so it was removed from the model.

*Supplementary Table S7* Results of the full model testing the effects of familiarity, valence, sex, and age on temperature change at the nose tip at 200 seconds

|  | Estimate | S.E. | t value | LRT | P |
| --- | --- | --- | --- | --- | --- |
| (Intercept) | *1.769* | *0.747* | *2.366* |  |  |
| Familiarity (Unfamiliar)^a^ | 0.058 | 0.176 | 0.331 | 0.111 | 0.739 |
| Valence (Neutral) | -0.451 | 0.242 | -1.861 | 3.460 | 0.063 |
| Age | -0.827 | 0.418 | -1.978 | 4.264 | **0.039** |
| Sex (Male) | 0.384 | 0.205 | 1.872 | 3.631 | 0.057 |

Significant values (P<0.05) are in **bold;** almost significant values are underlined (P < 0.9). S.E.: standard error; LRT: Likelihood Ratio Test; P: p-value. ^a^, The familiarity-valence interaction term was not significant, so it was removed from the model.

*Supplementary Table S8* Results comparing full and null models for IRT (thermal) and behavioural variables

| **Response variables: change in skin temperature relative to baseline, from stimulus onset** | **χ2** | **d.f.** | **p** |
| --- | --- | --- | --- |
| peri orbital region 80 sec | 1.976 (1.964) | 5 (4) | 0.852 (0.742) |
| peri orbital region 200 sec | 4.697 (2.439) | 5 (4) | 0.454 (0.656) |
| nose bridge region 80 sec | 9.727 (1.639) | 5 (4) | 0.083 (0.802) |
| **nose bridge region 200 sec** | 12.591 (11.579) | 5 (4) | **0.027 (0.021)** |
| nose tip region 80 sec | 8.055 (2.135) | 5 (4) | 0.153 (0.711) |
| **nose tip region 200 sec** | 11.067 (10.043) | 5 (4) | 0.050 **(0.040)** |
| **Response variables: behaviours** | ***χ2*** | ***d.f.*** | ***p*** |
| Stimulus avoidance (duration) | 3.746 (2.948) | 5 (4) | 0.587 (0.567) |
| Concern facial expressions (duration) | 7.739 (3.878) | 5 (4) | 0.171 (0.422) |
| Concern facial expressions (binomial) | 2.496 (3.013) | 5 (4) | 0.777 (0.556) |
| Other negative facial expressions (duration) | 6.095 (0.674) | 5 (4) | 0.297 (0.674) |
| Positive facial expressions (duration) | 8.672 (8.326) | 5 (4) | 0.122 (0.080) |
| Self-directed behaviours (duration) | 4.158 (4.153) | 5 (4) | 0.527 (0.386) |
| Pointing (occurrences) | 8.328 (8.245) | 5 (4) | 0.139 (0.083) |

Significant values (P<0.05) are in **bold;** almost significant values are underlined (P < 0.9); in brackets are the result from full-null comparison without the interaction between familiarity and valence

**Model results for positive expressions**

For positive expressions, the full model was only marginally better than the null model (*χ*^2^=8.33, d.f. = 4, *p* = 0.080), therefore model results are treated with caution. The Familiarity*Valence interaction was not significant; therefore it was removed. As predicted, the production of positive facial expressions increased more strongly in the neutral condition compared to the distress condition, relative to baseline (estimate ± se = 2.43 ± 1.19, t = 2.04, *p* = 0.045). There was no effect of familiarity of the stimulus, nor age or sex of the participant (Table S9 and Figure S1).

Although children produced fewer positive facial expressions when watching the distress condition than the neutral condition, some positive facial expressions did still occur in this context. Production of positive expressions in the face of a stressor has been indicated as a potential regulatory coping mechanism, with positive facial expressions like smiling suggested to lower physiological arousal in negative situations (Levenson et al., 1990), and socially engage with others (Fredrickson & Levenson, 1998; McEwen, 1993).


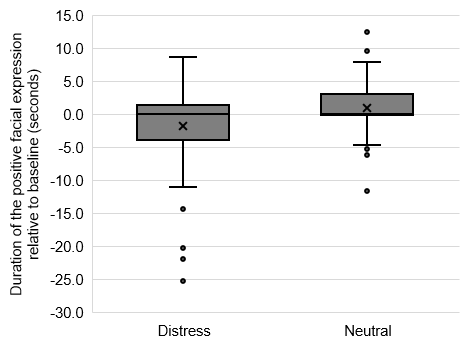


*Figure S1* Production of positive facial expressions as a function of the valence of the stimulus. The bars across boxes are medians; crosses represent means. Dots represent outlier data that are outside the 1.5 times the IQR, up and down.

*Supplementary Table S9* Results of the full model testing the effects of familiarity, valence, sex, and age on the production of positive facial expressions

|  | Estimate | S.E. | t value | LRT | P |
| --- | --- | --- | --- | --- | --- |
| (Intercept) | 5.707 | 4.605 | 1.239 |  |  |
| Familiarity (Unfamiliar)^a^ | -1.274 | 0.794 | -1.604 | 2.668 | 0.102 |
| Valence (Neutral) | 2.431 | 1.192 | 2.040 | 4.031 | **0.045** |
| Age | -3.786 | 2.554 | -1.482 | 2.367 | 0.124 |
| Sex (Male) | -0.006 | 1.258 | -0.005 | 0.009 | 0.924 |

Significant values (P<0.05) are in **bold.** S.E.: standard error; LRT: Likelihood Ratio Test; P: p-value. ^a^ The familiarity-valence interaction term was not significant, so it was removed from the model.

**Supplementary References**

Brody, L. (1999). *Gender, Emotion, and the Family*. Harvard University Press.

Campbell, R., Elgar, K., Kuntsi, J., Akers, R., Terstegge, J., Coleman, M., & Skuse, D. (2002). The classification of ‘fear’ from faces is associated with face recognition skill in women. *Neuropsychologia*, *40*(6), 575–584. https://doi.org/10.1016/S0028-3932(01)00164-6

Chaplin, T. M. (2015). Gender and Emotion Expression: A Developmental Contextual Perspective. *Emotion Review*, *7*(1), 14–21. https://doi.org/10.1177/1754073914544408

Collignon, O., Girard, S., Gosselin, F., Saint-Amour, D., Lepore, F., & Lassonde, M. (2010). Women process multisensory emotion expressions more efficiently than men. *Neuropsychologia*, *48*(1), 220–225. https://doi.org/10.1016/j.neuropsychologia.2009.09.007

Fredrickson, B. L., & Levenson, R. W. (1998). Positive Emotions Speed Recovery from the Cardiovascular Sequelae of Negative Emotions. *Cognition and Emotion*, *12*(2), 191–220. https://doi.org/10.1080/026999398379718

Hall, J. A. (1978). Gender Effects in Decoding Nonverbal Cues. *Psychology Bulletin*.

Hampson, E., van Anders, S. M., & Mullin, L. I. (2006). A female advantage in the recognition of emotional facial expressions: Test of an evolutionary hypothesis. *Evolution and Human Behavior*, *27*(6), 401–416. https://doi.org/10.1016/j.evolhumbehav.2006.05.002

Han, S., Fan, Y., & Mao, L. (2008). Gender difference in empathy for pain: An electrophysiological investigation. *Brain Research*, *1196*, 85–93. https://doi.org/10.1016/j.brainres.2007.12.062

Izard, C. E. (1979). The maximally discriminative facial movement coding system (MAX). *Newark, DE: University of Delaware Instructional Resources Center*.

Kring, A. M., & Gordon, A. H. (1998). Sex differences in emotion: Expression, experience, and physiology. *Journal of Personality and Social Psychology*, *74*(3), 686–703. https://doi.org/10.1037/0022-3514.74.3.686

Levenson, R. W., Ekman, P., & Friesen, W. V. (1990). Voluntary Facial Action Generates Emotion-Specific Autonomic Nervous System Activity. *Psychophysiology*, *27*(4), 363–384. https://doi.org/10.1111/j.1469-8986.1990.tb02330.x

McEwen, B. S. (1993). Stress and the Individual: Mechanisms Leading to Disease. *Archives of Internal Medicine*, *153*(18), 2093. https://doi.org/10.1001/archinte.1993.00410180039004

Thayer, J., & Johnsen, B. H. (2000). Sex differences in judgement of facial affect: A multivariate analysis of recognition errors. *Scandinavian Journal of Psychology*, *41*(3), 243–246. https://doi.org/10.1111/1467-9450.00193

Williams, L. M., Gatt, J. M., Hatch, A., Palmer, D. M., Nagy, M., Rennie, C., Cooper, N. J., Morris, C., Grieve, S., Dobson-Stone, C., Schofield, P., Clark, C. R., Gordon, E., Arns, M., & Paul, R. H. (2008). The integrate model of emotion, thinking and self regulation: An application to the ‘paradox of aging’. *Journal of Integrative Neuroscience*, *07*(03), 367–404. https://doi.org/10.1142/S0219635208001939
